# Coordination of Growth, Chromosome Replication/Segregation, and Cell Division in *E. coli*

**DOI:** 10.3389/fmicb.2018.01469

**Published:** 2018-07-09

**Authors:** Nancy E. Kleckner, Katerina Chatzi, Martin A. White, Jay K. Fisher, Mathieu Stouf

**Affiliations:** ^1^Department of Molecular and Cellular Biology Harvard University, Cambridge, MA, United States; ^2^Redbud Labs, Durham, NC, United States

**Keywords:** *E. coli*, cell cycle coordination, bacteria, chromosome, cell division, DNA replication, licensing

## Abstract

Bacterial cells growing in steady state maintain a 1:1:1 relationship between an appropriate mass increase, a round of DNA replication plus sister chromosome segregation, and cell division. This is accomplished without the cell cycle engine found in eukaryotic cells. We propose here a formal logic, and an accompanying mechanism, for how such coordination could be provided in *E. coli*. Completion of chromosomal and divisome-related events would lead, interactively, to a “progression control complex” (PCC) which provides integrated physical coupling between sister terminus regions and the nascent septum. When a cell has both (i) achieved a sufficient mass increase, and (ii) the PCC has developed, a conformational change in the PCC occurs. This change results in “progression permission,” which triggers both onset of cell division and release of terminus regions. Release of the terminus region, in turn, directly enables a next round of replication initiation via physical changes transmitted through the nucleoid. Division and initiation are then implemented, each at its own rate and timing, according to conditions present. Importantly: (i) the limiting step for progression permission may be either completion of the growth requirement or the chromosome/divisome processes required for assembly of the PCC; and, (ii) the outcome of the proposed process is granting of permission to progress, not determination of the absolute or relative timings of downstream events. This basic logic, and the accompanying mechanism, can explain coordination of events in both slow and fast growth conditions; can accommodate diverse variations and perturbations of cellular events; and is compatible with existing mathematical descriptions of the *E. coli* cell cycle. Also, while our proposition is specifically designed to provide 1:1:1 coordination among basic events on a “per-cell cycle” basis, it is a small step to further envision permission progression is also the target of basic growth rate control. In such a case, the rate of mass accumulation (or its equivalent) would determine the length of the interval between successive permission events and, thus, successive cell divisions and successive replication initiations.

## Introduction and overview

All cells growing in steady state must ensure a 1:1:1 relationship among doublings of cell mass, rounds of chromosome duplication/segregation and cell divisions. In eukaryotic cells, this relationship is ensured by operation of the cell cycle engine, in interplay with affected molecular events (Siddiqui et al., 2013). How this relationship is ensured in prokaryotes, e.g., *E. coli*, is unclear but widely discussed. Notably, *E. coli* can grow with both linear and overlapping chromosome/division cycles, with wide variations in the durations of component processes in different situations and, in any given situation, among different individual cells. The need for a coherent coordination process seems especially important in light of this dramatic variability on both the population and single cell levels. Here we propose that a process exists specifically to ensure the necessary 1:1:1 coordination and we propose both a formal logic and a specific mechanism for such coordination. Furthermore, we suggest that the proposed process could serve not only for coordination, but also as the mechanism by which occurrence of cell division [and an accompanying round of initiation(s)] is linked to cell growth conditions.

**Figure 1:**
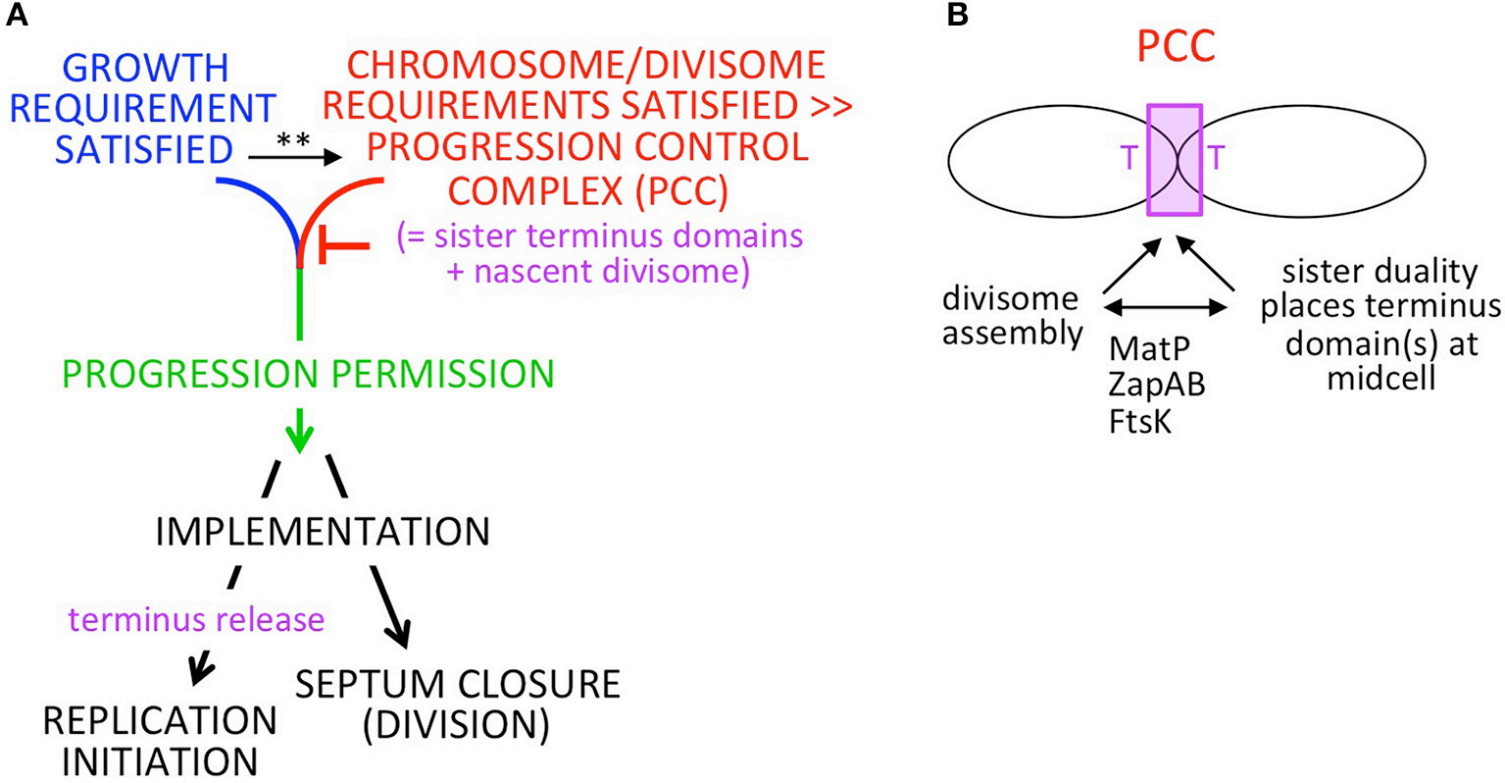
Progression permission model. **(A)** General logic for 1:1:1 coordination of cell growth, replication initiation and cell division. Note: in slow growth conditions, PCC development clearly precedes satisfaction of the growth requirement such that the two features operate in parallel. In fast growth conditions, it is less clear whether the growth input is independent of PCC development and/or feeds into development of the PCC. This ambiguity is indicated by the (^**^); see text. **(B)** Development of the proposed PPC by integration of chromosome and divisome inputs.

In brief: (Figure 1A), when the cell has satisfied requirements both for growth (mass accumulation or its correlate) and for completion of chromosome replication/segregation and divisome development (which are functionally related processes; below), chromosomal events and septum closure are coordinately permitted to progress, resulting in, respectively, replication initiation and cell division. After progression permission has been granted, the two downstream outcomes will be implemented. This formal logic will function regardless of which of the two required input events is rate-limiting. During implementation, the absolute and relative timings of the two downstream outputs will be influenced by the rates of individual component events. We show below that this logic can function analogously in slow and fast growth regimes; that it is robust to variations in the rates cellular events; and that it can gracefully accommodate growth rate transitions.

A key feature of the proposed mechanism for this process is a progression control complex (PCC) (Figure 1A). This PCC would form by interaction of sister terminus domains with the developing mid-cell divisome, dependent on proteins known to interactively mediate chromosome/divisome interplay (Figure 1B). Once formed, the PCC would inhibit onset of a next round of replication initiation and onset of cell division. Concomitantly, growth-related events are occurring.

In some situations (e.g., slow growth conditions), completion of the growth requirement will be rate-limiting irrespective of chromosome/divisome events, with PCC-mediated inhibition remaining in play until the growth requirement is met. In other situations (e.g., fast growth conditions), the chromosome/divisome events involved in PCC development seem to be rate limiting. In these conditions, it is less clear when and how the sensing of growth status occurs and thus this input may be independent of PCC development or feed directly into PCC development itself (or potentially both) (Figure 1A legend). In any of these cases, however, progression permission would occur as soon as PCC development is complete.

In all growth conditions, once both the growth and chromosome/divisiome requirements have been met, the PCC would undergo a conformational change that concomitantly: (i) triggers onset of septum closure (and thus cell division); and (ii) releases the terminus domain from divisome components (and thereby allowing a next round of replication initiation to occur whenever other requirements and required components are present). This conformational change in the PCC would comprise “progression permission” (Figure 1A green).

We further suggest that the PCC transition that triggers resultant division/initiation could be the event by which cells sense and respond to growth condition, with PCC transition events occurring more or less frequently under faster or slower growth conditions.

We also note that, as described below, the mechanism described for these effects involves not only direct physical interaction among relevant components but a physical mechanism for constraining and permitting replication initiation that involves transmission of information throughout the nucleoid. Such a process would be an attractive way to achieve coordination (and control) in the absence of a eukaryotic-like cell cycle engine.

## Formal logic

*E. coli* grows in two different regimes, termed “slow” and “fast” growth (e.g., Helmstetter et al., 1968; Wallden et al., 2016).

**Figure 2:**
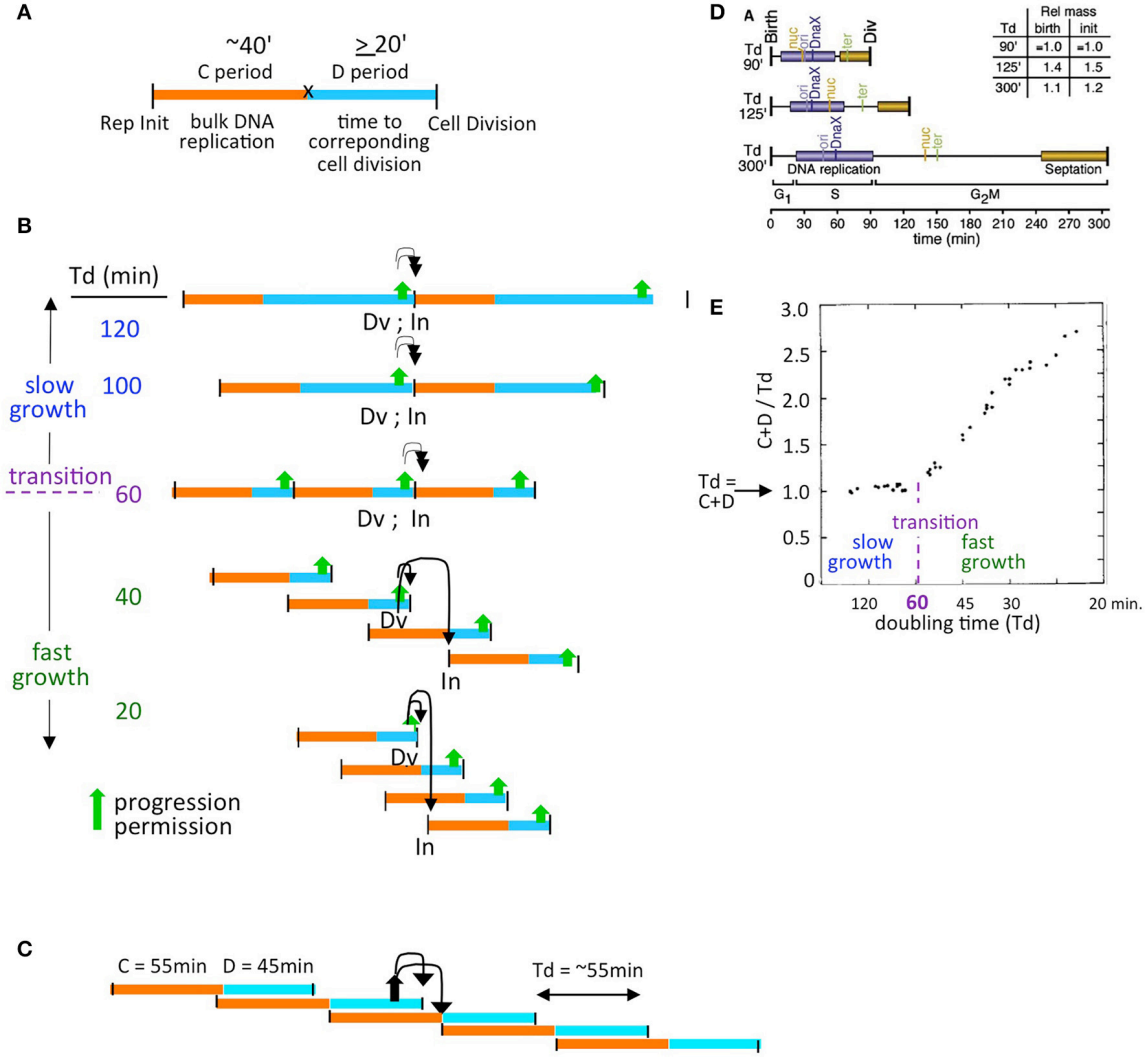
The proposed model is compatible with diverse growth conditions. **(A)** Definition of classical Cooper-Helmstetter C and D periods (Helmstetter et al., 1968). **(B)** Natures of, and relationships among, slow and fast growth in terms of (C+D) sequences and the effect of coordination control in the two situations. Progression permission is indicated by a filled upward arrow; downward arrows indicate the corresponding permitted cell division and replication initiation. **(C)** (C+D) sequences for an *E. coli* K12 strain with non-canonical period lengths [Td = 55 min; (C+D) = 100 min, with a 55 min C period and a 45 minute D period; Nielsen et al., 2007]. **(D)** Synchronous cell analysis of chromosome and divisome events under several slow growth conditions show that replication (purple) always begins soon after division (from Bates and Kleckner, 2005). **(E)** Relationships between the length of (C+D) and doubling time (Td) as a function of doubling time (from Helmstetter et al., 1968).

One approach to describing these regimes is provided by the formulation of Cooper and Helmstetter, who divided the cell division cycle into two components - the “C-period,” which comprises bulk DNA replication, and the “D-period” which comprises the period between the end of bulk DNA replication and the corresponding division, at which the sisters generated during replication segregate to daughter (sister) cells (Helmstetter et al., 1968; Figure 2A).

Cooper and Helmstetter's classical studies used strain B/r, where the C period is roughly constant at ~40 min under diverse conditions.

- In “slow growth” conditions, C and D periods follow one another in sequence, with a constant C-period followed by an appropriately long D-period. At progressively shorter doubling times, the D-period becomes progressively shorter (Figure 2B top).
- At a certain doubling time (classically ~60 min, implying a D-period of ~20 min) a minimum length of the D-period is reached (Figure 2B middle). This condition marks the transition between slow and fast growth. The basis for this minimum D-period is not established; however a strong possibility is that a certain minimum time is required for completion of sister terminus separation (e.g., dimer reduction and decatenation) and for the actual act of septum formation via closure of the Z-ring, which are two closely interrelated processes.
- Thereafter, in the “fast growth” regime, cells can double more often than every 60 min, but now do so via partially overlapping (C+D) periods, with such periods occurring at intervals corresponding to the mass doubling time (Figure 2B bottom).

The same rules pertain analogously in *E. coli* K12, which exhibits a diversity of C and D period lengths under different conditions (e.g., Figure 2C).

Experimentally, it is observed that, in slow growth conditions, each cell division is closely accompanied by a next round of replication initiation (e.g., Bates and Kleckner, 2005; Figure 2D), with the two events occurring in either order according to the situation. Close coupling of division and initiation is also a necessary consequence of the Cooper-Helmstetter formalism because, in slow growth conditions, (C+D) periods follow sequentially one upon the other (Figure 2A). Correspondingly, in these conditions, (C+D) is the same as the doubling time ((C+D)/Td = 1; Helmstetter et al., 1968; Figure 2E).

This sequence of events seen in slow growth conditions gives the impression that, following a division, the chromosome cycle is initiated and completed, and then the cell waits until it becomes large enough, at which time it divides. Put another way: it seem as if the timing of division (and an accompanying replication initiation) is limited by cell growth (although for an alternative, see Logsdon et al., 2017).

In contrast, in fast growth conditions, where (C+D) has reached its minimum value and the cell has been forced into overlapping (C+D) periods, it seems as if chromosome/divisome events are limiting.

Both situations are all accommodated by the formal logic of progression permission control described above (Figures 1A, 2B,C). A cell must satisfy both its growth requirement and its chromosome/divisome requirements in order to progress to the next round of cell division and replication initiation.

In Cooper-Helmstetter terminology, we thus envision that each particular (C+D) sequence sets up a PCC. Then, once the two requirements of growth and PCC formation are satisfied, progression permission would occur. This event will always enable occurrence of the division that defines the end of the initiating (C+D) sequence and will also enable occurrence of a next round of replication initiation (which may then occur sooner or later according to the conditions). In slow growth, this sequence of events leads to one division and an accompanying replication initiation on each of the single sister chromosomes in each daughter cell (Figure 2B).

In fast growth, this leads to a division and a round of replication initiations that occur on all origins present in the two daughter cells at that particular time (Figure 2B), in accord with the fact that all of the cell's origins fire synchronously under fast growth conditions (Skarstad et al., 1986).

This basic logic pertains to diverse “wild type” growth conditions regardless of the exact lengths of the C and D periods, which are known to vary widely among different *E. coli* strains and conditions (e.g., Nielsen et al., 2007; Figure 2C; below).

### Mechanism: insights from slow growth

The notion of a growth-sensitive progression permission process, and the above-proposed mechanism for such a process, have emerged from detailed analysis of events in slow growth conditions.

We previously observed, under such conditions, not only that division and replication initiation are closely coupled in time (above) but also that the process of division is accompanied by a change in the disposition of the nucleoid which, in turn, precedes initiation of DNA replication. Importantly, cell division and this nucleoid transition occur independently of one another. This feature suggests that the two events could be parallel downstream outcomes of a common upstream event. Given that accumulation of sufficient cell mass triggers onset of division (above), these findings give rise to the simple notion that accumulation of sufficient cell mass triggers two coordinate events: (i) onset of septation; and (ii) a change in nucleoid state which, in turn, makes possible replication initiation. (For comments on this previously-proposed idea and its subsequent misinterpretations, see footnote^1^).

**Figure 3:**
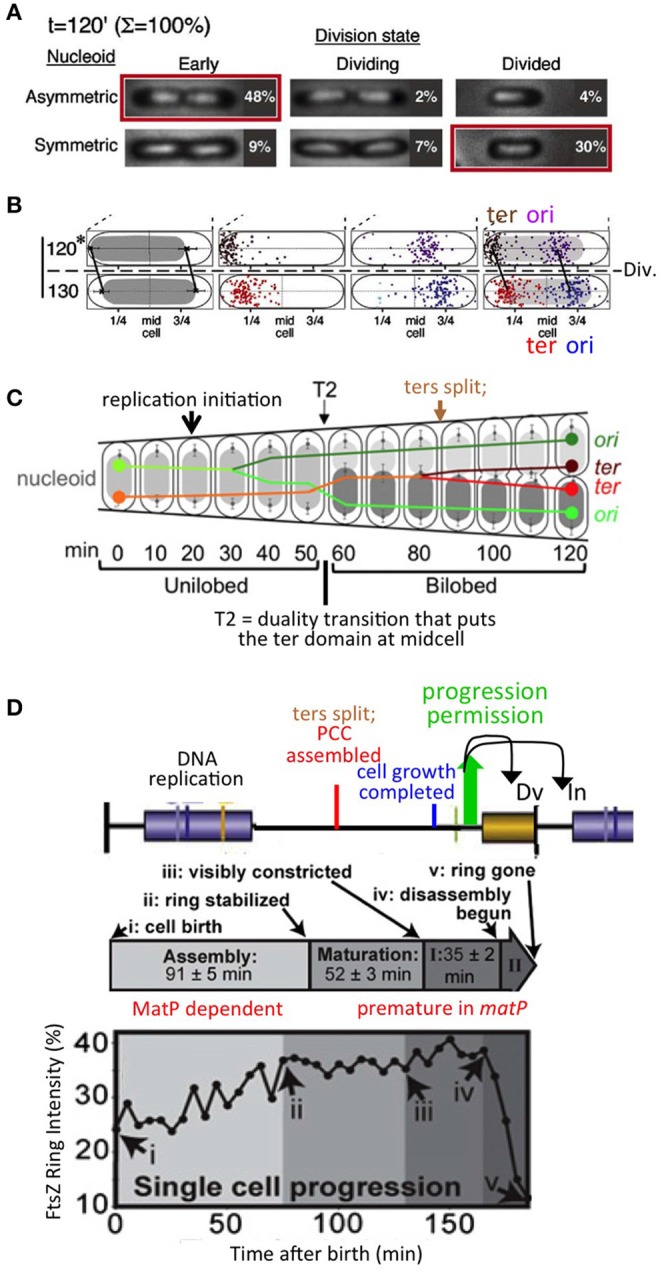
Chromosomal and divisome events under slow growth conditions. **(A)**. Cell division is accompanied by a change in nucleoid disposition, from sister nucleoids closely juxtaposed to mid-cell via their terminus regions to each nucleoid centrally positioned within its (future or existing) sister (daughter) cells. Note that septum closure and the nucleoid transition can occur in either order, implying that they are independent events. **(B)** The nucleoid transition in **(A)** involves a whole body movement of the nucleoid, with origin and terminus regions remaining in the same relative positions. **(A,B)** are from Bates and Kleckner (2005). **(C)** Sequence of chromosomal events including replication initiation; a prominent transition to nucleoid duality, accompanied by an exchange of places of one sister (marked by its origin) and the mother material (marked by its terminus), and ensuing terminus dynamics including splitting and transit of one terminus across midcell (from Joshi et al., 2011 based on data in Bates and Kleckner, 2005). **(D)**. Divisome (FtsZ) assembly dynamics defined under slow growth conditions (from Coltharp et al., 2016) (middle and bottom) as compared to chromosomal events predicted by interpolation of data from similar conditions (Figure 1D) and the proposed progression permission/PCC model (Figures 1A,B).

The specific observations that led to this idea are as follows (Bates and Kleckner, 2005; Figures 3A,B). Prior to onset of septation, sister nucleoids are closely juxtaposed to midcell via their terminus regions, and thus asymmetrically positioned within their respective emerging cells. Then, two events occur concomitantly: (i) the septum closes, thus implementing cell division; and (ii) the nucleoid is released from midcell, after which it comes to occupy a more central location within the cell (Figure 3A). This change in nucleoid position occurs without any change in the positions of the origin and terminus within the nucleoid, and thus appears to comprise a “whole body” movement of the nucleoid (Figure 3B). Importantly, since completion of septation and nucleoid release can occur in either order (Figure 3A), these two events are independent and thus could be parallel downstream outcomes of an earlier event (above). Moreover, following nucleoid release, the origin moves toward the middle of the cell, while the terminus region also moves inward, after which replication initiates (Figure 3C). Thus, release of the nucleoid from midcell could potentially permit initiation of replication.

This latter idea has the additional implication that establishment of tethering of the nucleoid to midcell poses a block to initiation, with initiation then permitted by release from that tethering. To explain how release of tethering might have this effect, we further suggested that the signals for blocking, and then permitting, replication initiation would involve propagation of a change in state through the nucleoid itself. The possibility of such an effect was raised originally by the finding, in this same study, that release of key sister linkages, now known to be mediated by long-lasting inter-sister “snaps,” results in a global reorganization of the nucleoid (Bates and Kleckner, 2005; Joshi et al., 2011; Figure 3C). This possibility was further supported by the discovery that the nucleoid is a physically coherent object, which appears to be intrinsically stiff (Wiggins et al., 2010; Hadizadeh Yazdi et al., 2012; Fisher et al., 2013), and which undergoes coherent motions along its entire length and width, on ~20 and ~2 min time scales (Fisher et al., 2013).

Subsequently, the tether/release idea was tested directly by Bates and colleagues, who examined the consequences of artificially tethering the nucleoid to the edge of the cell (Magnan et al., 2015). That study found that artificial tethering causes a block to replication initiation, but without blocking completion of ongoing replication, and moreover that this effect is accompanied by a global loss of negative supercoiling, which presumably explains the initiation block. These are exactly the types of effects originally envisioned.

From this and other information, we can now suggest a more complete mechanism for a regulatory process as outlined above (Figures 1A,B). Ultimately, a central feature is physical association between replicated sister terminus domains and the nascent mid-cell septum which emerges in a mutually inter-dependent process, giving the proposed PCC (Figure 1B). When requisite cellular events are completed, the PCC would undergo a conformational change that coordinately triggers onset of septation (and thus cell division) and release of the terminus domain (which, in turn, permits replication initiation). The conformational change in the PCC would comprise “progression permission.” Progression permission would then be followed by implementation of the two downstream outcomes, which occur on their own respective clocks, dependent on relevant conditions and factors. For example, it can be expected that alterations in initiation factors (e.g., Boye et al., 1996; Ryan et al., 2004; Kasho et al., 2014, 2017; Sakiyama et al., 2017; DnaA, IHF and supercoiling) may delay or accelerate the timing of replication initiation by effects on implementation, downstream of progression permission. The same considerations apply to cell division, which (like initiation) is sensitive to growth conditions (Coltharp et al., 2016). These effects can explain, for example, why initiation and division can occur in either order in slow growth conditions (above) and why the relative times of initiation and division are predicted to vary under different fast growth regimes (Figures 2B,C). Conversely, observation of such differences has no bearing on the validity of the proposed logic and mechanism (see Footnote^1^).

The existence of the proposed terminus domain/nascent divisome PCC is further supported by the following observations.

1. A key event described for slowly-growing cells is a global transition that places the terminus domain in the vicinity of mid-cell. In brief, sisters initially emerge to the same side of unreplicated mother material. At a certain point, a global transition occurs in which one sister (and its origin) changes places with the mother material, placing the terminus domain in the vicinity of midcell, after which it undergoes further changes, e.g., splitting and movement of one terminus to the opposite side of midcell, which presumptively reflect capture of the terminus domain by the divisome (Bates and Kleckner, 2005; Figure 3C). These events are completed well before division, more or less in the middle of the “D-period” (Figure 3C). We show below that an analogous effect occurs in fast growth conditions.
2. Analysis of septum-formation in slowly growing cells (Coltharp et al., 2016) shows that the amount of FtsZ at mid-cell increases, stabilizes and remains at a high level for a significant period of time until onset of septation. The time at which stabilization occurs is more or less in the middle of the “D-period,” i.e., in the same time window that terminus domain events are being completed (Figure 3D).
3. Functionally, the terminus domain is required for normal development of the septum: MatP, which is specifically devoted to terminus domain organization, is required for proper localization and development of the septum (Coltharp et al., 2016; Figure 1B). Absence of MatP has the same effects on mid-cell FtsZ accumulation as absence of MinC, a negative regulator of septum localization via the MinCDE system.
4. Moreover, and of especial importance: in the absence of MatP, both sister segregation and onset of septum formation are premature (Mercier et al., 2008; Coltharp et al., 2016). The latter finding led to the conclusion that chromosome segregation (along with cell wall synthesis) are rate-limiting for division. In the present context, this finding strongly suggests, directly, that the presence of the terminus domain is important for impeding, and then allowing proper timing of, septal ring closure for cell division, as we propose.
5. Association of the terminus region with the septum is well known to be important for proper completion of terminus-related events, including dimer reduction and decatenation (reviewed in Reyes-Lamothe et al., 2012). The terminus region and MatP interact with the developing septum via ZapAB (Männik et al., 2016; Buss et al., 2017). Moreover, FtsK is an intriguing candidate for a molecule that mediates signaling in both directions between the terminus domain and the septum, as in our proposed model. FtsK is in direct contact with both the cell septum and the chromosomal terminus region and is essential for execution of both cell division and regular chromosome segregation (reviews in Grainge, 2010; Bouet et al., 2014). Correspondingly, FtsK has already been proposed to be a mediator of coordination between final events of the chromosome cycle and cell division (Stouf et al., 2013), to contribute to nucleoid/septum localization (Bailey et al., 2014) and to sense addition of a fixed increment of cell size between divisions under fast growth conditions (Campos et al., 2014; below).
6. Events of the chromosome cycle can proceed efficiently in the absence of cell division (e.g., in *ftsZ* and *ftsK* mutants) and also even in the absence of a cell wall (in “L-forms”; K. Chatzi, M. Stouf, and N.K. unpublished). This fact implies that a specific mechanism for coordination of chromosomal events with divisome events must be essential for regular growth and division in wild type cells. We also note that our model specifically predicts that the chromosome cycle will run free in *ftsZ/ftsK* mutants: in the absence of FtsZ/K, no PCC will form and thus neither the inhibition nor the enabling of replication initiation will occur. [We also reiterate actually that division *per se* is not required for initiation of replication in our proposed scenario (Figure 1A), because septum closure and terminus release (and thus initiation) are observed to be parallel, independent events (Figure 3A) (see Footnote^1^).

### Mechanism: extension to fast growth

The scenario that emerges from analysis of slow growth conditions can be mapped analogously onto chromosomal/nucleoid and divisome events that occur under fast growth conditions. In principle, each replication initiation that marks the start of a C period should set up a corresponding sister ter/septum PCC (above; Figures 2B,C). Furthermore, PCC assembly is presumably always completed after completion of bulk DNA replication, i.e., during the ensuing D-period, also in slow growth conditions.

**Figure 4:**
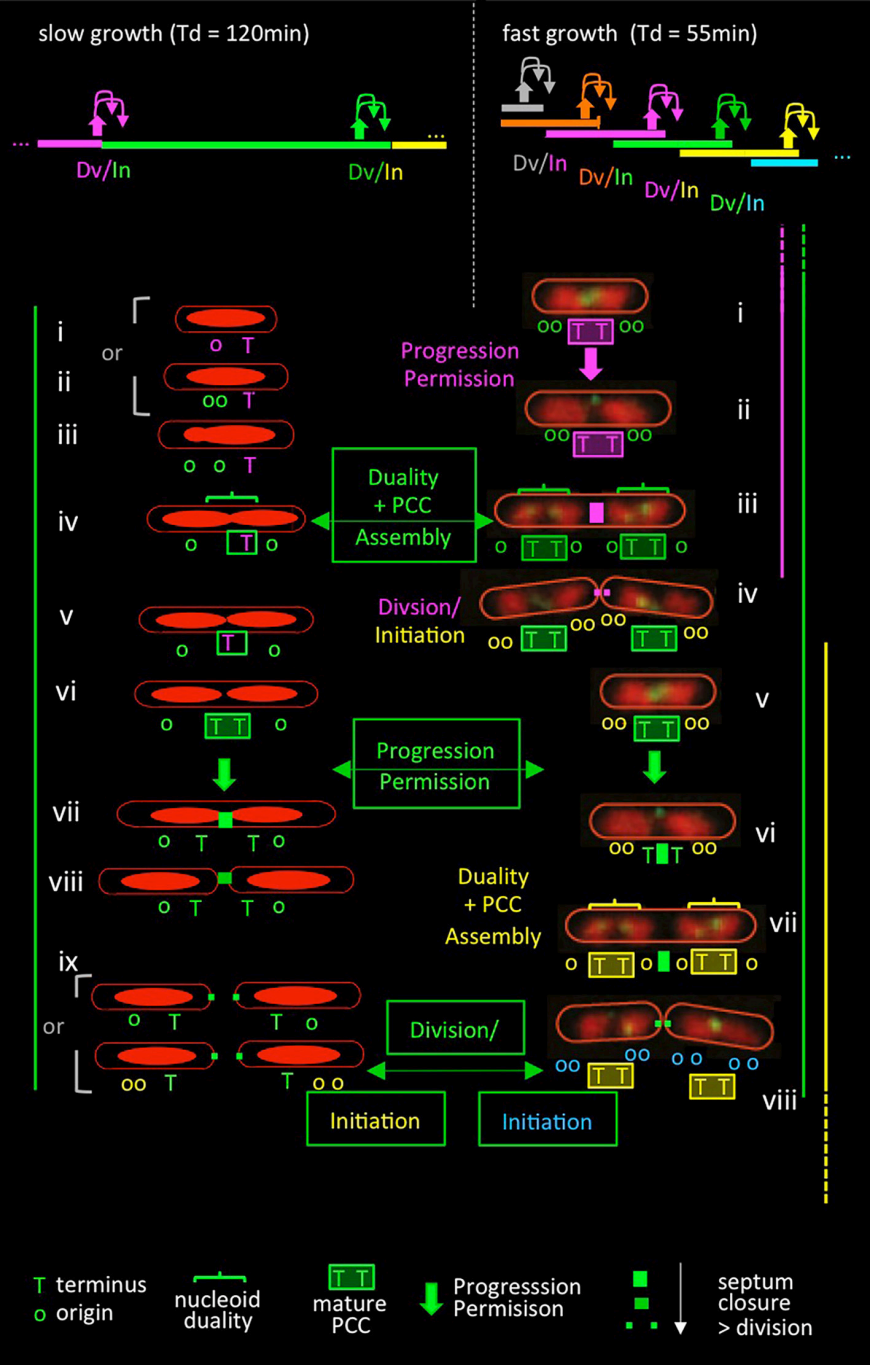
Comparison of events in slow and fast growth conditions in the context of the progression permission model. Top: patterns of (C+D) sequences (bar) with corresponding events of coordination control including progression permission (upward filled arrows) and the corresponding permitted cell division and replication initiation events (downward arrows). Slow growth patterns correspond to conditions in Figures 3A–C (Bates and Kleckner, 2005); fast growth patterns correspond to conditions in Figure 2C (Nielsen et al., 2007). Bottom: patterns of events in slow (left) and fast (right) growth conditions. Events are color-coded in relation to the (C+D) period to which they correspond, as defined in the top panel. Bottom left side: patterns of nucleoid morphologies and terminus and origin dynamics observed experimentally in slow growth conditions [(Bates and Kleckner, 2005); Figure 3C] plus predicted events of the proposed progression permission process including PCC assembly, progression permission, and the ensuing permitted division and replication initiation. Note that replication begins after division in the study of Bates and Kleckner (Figure 3) but often begins just before division in a number of other slow growth conditions. Bottom right side: nucleoid and terminus morphologies extracted from live cell time-lapse movies of Youngren et al. (2014) and overlaid with predicted events of the proposed progression permission process as it would occur in the corresponding partially overlapping (C+D) periods. Origin numbers and dispositions predicted from “C+D” patterns (Nielsen et al., 2007) are superimposed. Events in slow and fast growth conditions are directly compared by the (C+D) sequences defined in green boxes, as described in the text. The replication initiations resulting from these sequences are shown at the bottom in green boxes, with origin colors of the corresponding (C+D) sequence. (Note that somewhat different replication timing was inferred by analysis of fluorescent foci of SSB; however, that inference failed to take into account the fact that sister replisomes tend to first cluster and then split, implying that SSB foci are not a reliable indicator of the number of replication forks. Indeed, data inspection shows that pairs of SSB foci tend to emerge at the same time as nucleoid duality, in accord with occurrence by splitting rather than as a reflection of the time of initiation).

As described for slow growth conditions above, key events for establishment of the PCC should be: (i) a duality transition that places sister terminus domains at mid-cell and (ii) interaction of the terminus region specifically with the septum. Then: a conformational change in the PCC should permit both onset of septation and terminus release (and thereby replication initiation) (cartoon in Figure 4 left, top and bottom).

Evidence that exactly the same progression occurs in fast growth conditions can be obtained by appropriate inspection of time-lapse data for doubling every ~55 min (Youngren et al., 2014; Figure 2C; Figure 4 right, top and bottom).

About-to-divide (or divided and unseparated) cells have four nucleoid units, with a set of two units located on either side of the division site, and with a pair of terminus regions located at the inner borders between each pair (Figure 4 right, iv and viii). This configuration persists for a while after division. Thus, each daughter cell contains two now-expanding nucleoid bodies with a pair of terminus regions located between them, which position is also now midcell, and thus is the site of the next division (Figure 4 right, i,ii and v,vi). Then, each of the two terminus markers moves away from that pre-division site to a position within its respective adjacent nucleoid (Figure 4 right, ii to iii; vi to vii). This terminus transition has been strongly emphasized (Youngren et al., 2014). In the present context, it can be seen to be analogous to the division-associated event seen in slow growth conditions at which each sister terminus domain moves away from mid-cell to an inward position within its corresponding nucleoid (Figure 4 left, ix to i, ii). Moreover, in fast growth, this terminus transition is again followed shortly by division and, by the predictions of defined (C+D) periods (Figure 2C, Figure 4 legend), a round of replication initiations (Figure 4 right, iv and viii).

Detailed inspection of these fast growth images further reveals that movement of each terminus to within its adjacent nucleoid unit is accompanied by acquisition of duality within that unit (Figure 4 right, iii and vii, horizontal brackets) such that the terminus region is now located in between a pair of newly-individualized nucleoid units (Figure 4 right, iii and vii). The combination of nucleoid duality and placement of the terminus between the resultant pair of units corresponds to the critical duality transition seen in slow growth conditions where the terminus becomes localized between sister nucleoid units as part of a global reorganization (Figures 3C, 4 left, iii to iv).

Thus, under fast growth conditions, just as in slow growth conditions: (i) a transition occurs in which terminus domains are initially tethered to a future septum site and then released (analogously to events at the end of the slow growth program) and (ii) a nucleoid duality transition occurs that places a single terminus domain between two developing sister nucleoids (analogously to the duality transition that occurs in the middle of the slow growth program).

When these and other events are mapped onto the fast growing cell's multiple overlapping (C+D) periods (Figure 4 right), it emerges that: (i) the release of terminus domains from the future mid-cell division site should be the downstream outcome of a prior progression permission event (which concomitantly can be inferred to yield a round of replication initiation); and (ii) the nucleoid duality transition with accompanying inter-nucleoid unit terminus localization, should be the event that permits development of the PCC that mediates the next set of division and initiation events (Figure 4 right). When these events are viewed in the context of a single (C+D) period, they are exactly analogously to the sequence defined for slow growth conditions (e.g., Figure 4 left green with Figure 4 right green).

We also note that in this fast growth condition, a particular (C+D) sequence (e.g., C+D_N_; Figure 4 top right green) is initiated by progression permission two sequences earlier (C+D_N−2_; Figure 4 top right orange) and permits its corresponding division and a round of initiation for two sequences later (C+D_N+2_; Figure 4 top right turquoise), thereby spanning a total of five (C+D) sequences.

The same principles also explain *E. coli* K12 data obtained under very fast growth conditions. In cells growing with a doubling time of 17 min, about-to-divide cells contain 16 origins and four termini that are organized into four nucleoid units which, upon division, give cells with 8 origins, which are organized into two nucleoid units, each with a pair of termini (M. White and D. Leach, personal communication). Strikingly, these nucleoid configurations are essentially identical to those seen in cells doubling at a 55 min doubling time (above) and thus can be gracefully explained by simply adding another round of replication initiation to each nucleoid unit. Correspondingly, application of the above-described duality > PCC formation > progression permission pattern to this situation results in exactly the appropriate outcome, with each (C+D) sequence now spanning seven additional (C+D) sequences, i.e., (C+D_N−3_) to (C+D_N+3_), rather than the five observed at Td = 55 min (not shown).

That is: a simple progression defined by analysis of slow growth conditions can be mapped directly onto events of fast-growing cells despite the complexity conferred by multiple overlapping (C+D) sequences.

## Accommodations and compatibilities

The proposed coordination model directly accommodates natural variations in the lengths of the C and D periods (e.g., Figure 2C vs. Figure 2B).

**Figure 5:**
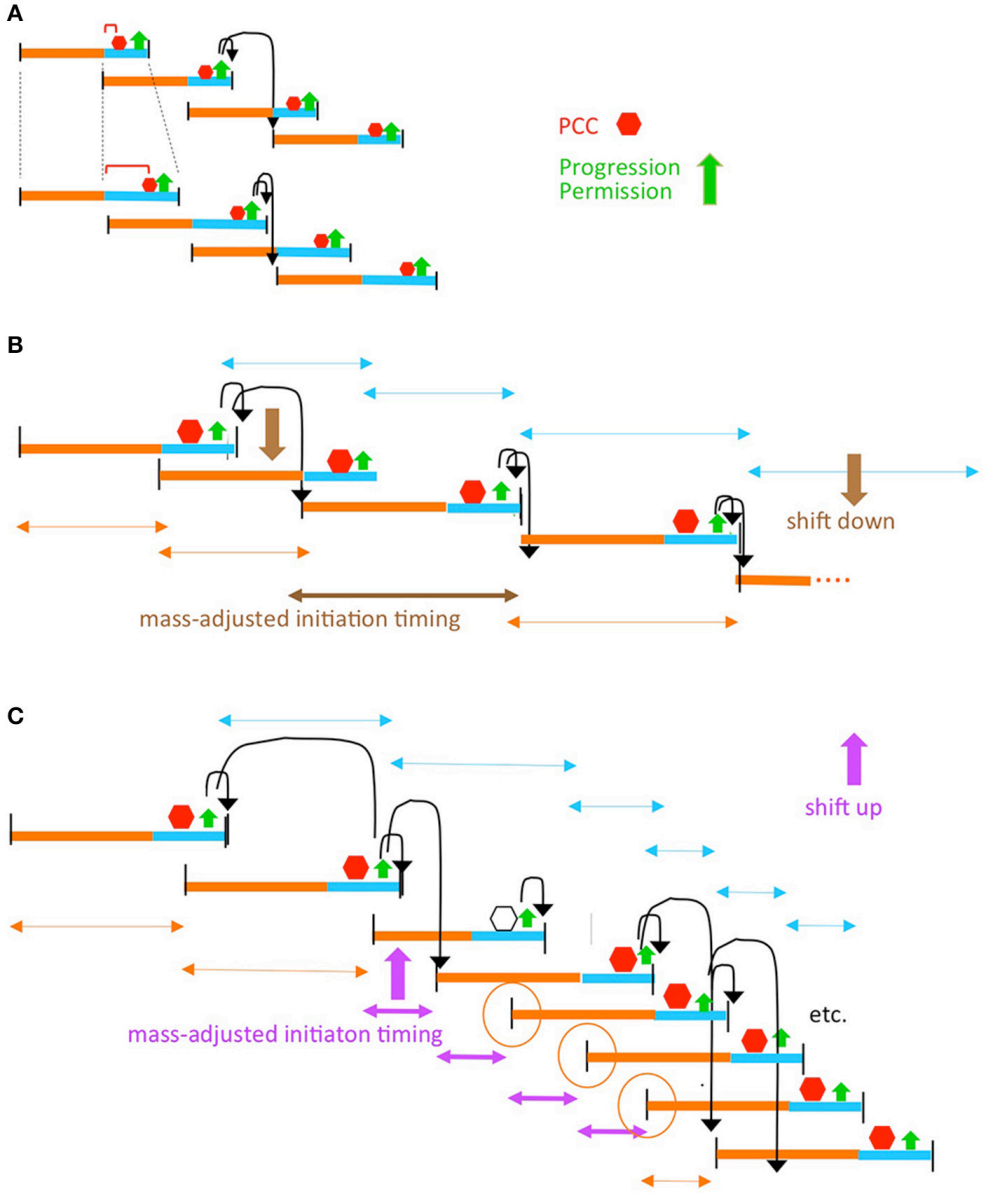
The progression permission model can accommodate diverse situations. **(A)** Perturbations of chromosome/divisome events that delay PCC formation. **(B)** Growth rate transitions. It is well established that, in a given growth condition, replication initiation tends to occur at a particular cell mass (sometimes parameterized as the mass/origin ratio; Donachie, 1968). Thus, in some situations, a change in growth conditions can be implemented by the simple expedient of having replication initiation occur at the cell mass corresponding to the new growth rate **(B)**. However, some situations, notably a dramatic increase in growth rate, require that replication initiate before the time at which it would normally be allowed to occur by a scheduled progression permission event. In such cases, the required adjustment can be made if PCC activity is compromised in such a way that it still forms in response to onset of a (C+D) sequence, and regulates the ensuing division, but is no longer able to regulate replication initiation. As a result, initiation can run free until such time as a properly constituted PCC has again formed **(C)**. Open hexagon indicates the (C+D) period in which PCC control over replication intiation is abrogated. Orange circles denote the replication initiations that are determined independently of PCC control due to the combined effects of PCC control abrogation and timing relative to re-establishment of PCC control. This scenario corresponds to the Cooper-Helmstetter observations that ongoing C period(s) is/are completed before there is a change to a new interval between divisions [the phenomenon of “rate maintenance” Helmstetter et al., 1968]; compare orange and turquoise double-headed arrows in **(B,C)**.

This model also accommodates the occurrence defects or delays in upstream processes required for the PCC transition. For example, certain mutations that affect chromosome state and/or divisome state can prolong the length of the D-period, without changing growth rate or the C period (Zheng et al., 2016; Si et al., 2017). Some or all of these mutations could confer their effects by altering the time required for development of the PCC (Figure 5A). Similarly, mutations that directly or indirectly reduce the rate of DNA replication will also delay PCC development, while retaining proper coordination, e.g., as in strains with naturally longer C-periods. In the extreme, such an effect could also explain why, in *dnaA* mutants and an initiation-specific *dnaC* mutant, cell division is blocked or delayed/defective, respectively (Cambridge et al., 2014; D. Bates, personal communication; Kleckner laboratory unpublished). In other situations, events related to sensing the accumulation of cell mass might be defective, again resulting in a delay in the PCC transition, but without loss of cooordination.

The proposed coordination model can also gracefully accommodate transitions from one growth rate to another (Figures 5B,C). Notably, dramatic upshifts can be mediated by differential disabling of the PCC(s) ability to regulate replication initiation (Figure 5C).

We also note that that all of the proposed effects pertain in the context of Cooper-Helmstetter formalism, as shown above. They are therefore compatible with mathematical descriptions that utilize that formalism. Most specifically, the exact timing of replication initiation could still be determined by, or at least closely correlated with, mass-to-origin ratio [“initiation mass”; Donachie 1968].

We also note that the proposed mechanism is logically distinct from canonical “checkpoint” mechanisms (e.g., Boye and Boye and Nordström, 2003; Arjes et al., 2014), although the coordination outcome is similar.

Finally, the possibility of a PCC-mediated coordination process also raises new possibilities for the origins of cell-to-cell variability. For example, an individual cell may sometimes divide without an initiation (Ho and Amir, 2015) or, oppositely, may undergo two initiations between cell divisions rather than one (Wallden et al., 2016). Analogous cell-to-cell variability occurs in the timing of cell division. In the context of the current model, they could be explained by stochastic fluctuations in upstream component events required for permission granting; in the actual PCC transition *per se*; and/or in the execution of events during the “implementation” stage.

## Does the PCC transition integrate cellular events with growth conditions?

The discussions above consider the issue of coordination, where assembly of the PCC and growth inputs lead to progression of the PCC in a process that acts to ensure a 1:1:1 relationship among the mass/size increase required for division, replication initiation/segregation and cell division *per se*.

**Figure 6:**
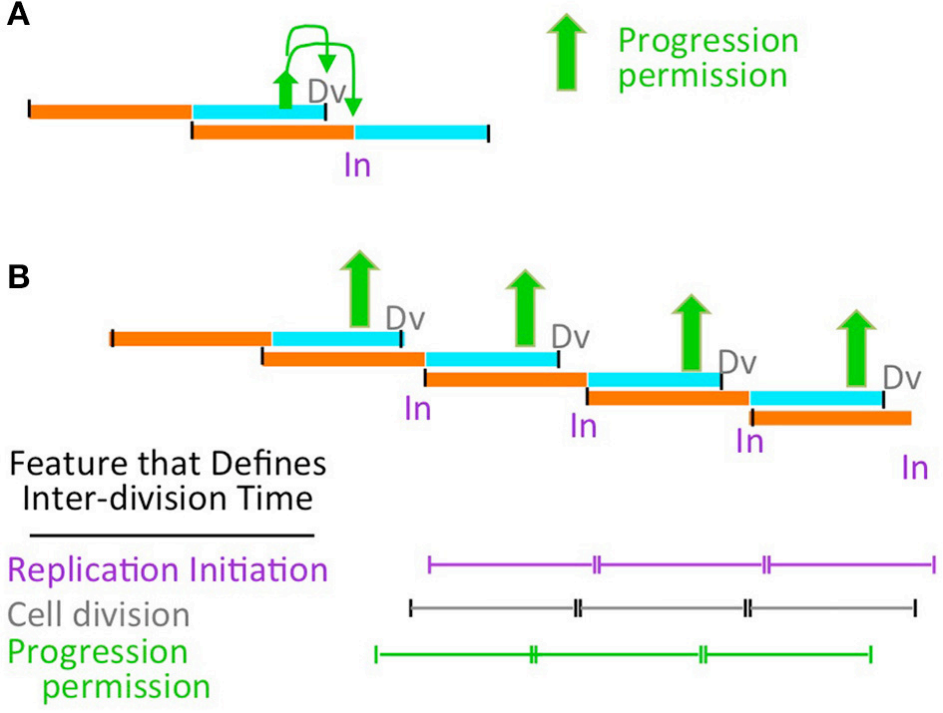
Possible relationship of progression permission to growth rate control. **(A)** Progression permission enables division and replication initiation in a particular growth regime. **(B)** In a given balanced growth condition, all indicated events occur in a 1:1:1:1:1 relationship with a particular relative timing (on a population average level). Thus, the ultimate determinant of cell division timing (e.g., by addition of a particular amount of cell mass Ho et al., 2018) could be progression permission (this work); cell division (Harris and Theriot, 2016) or replication initiation (Amir, 2017).

However, we suggest that this process is, in fact, the mechanism that links all three processes to cell growth conditions. In faster or slower growing cells, the cohort of coordinated events must occur more or less frequently. In its formal logic, our proposal is agnostic with respect to how this frequency is determined, i.e., in how occurrence of a cohort of coordinated events is coupled to the rate of cell growth (Figure 6).

Correspondingly, the above proposal is fully compatible with a process in which growth conditions are sensed at the replication initiation step, with all other events occurring as a downstream consequence, and without any input from cellular parameters other than cell mass (Figure 6 purple; Amir, 2017). Our proposal is similarly compatible with the proposition that inter-division time is sensed by properties of the cell container alone, e.g., surface to volume ratio, without considering input from chromosomal events (Figure 6 gray; Harris and Theriot, 2016).

On the other hand: it is straightforward to envision that interplay of growth with the PCC to give permission granting is actually the critical event that couples replication initiation and cell division to growth rate. In such a case, the rate of cell growth would determine the length of time between such transitions and, thereby, the inter-division time, with each transition resulting coordinately in both cell division and initiation of the next chromosome cycle (Figure 6 green).

Additionally, recent studies suggest that under fast growth conditions, division is triggered by accumulation of an appropriate fixed amount of cell length (or volume/mass) (the so-called “adder” rule; review in Ho et al., 2018). Under such conditions, acquisition of this fixed amount would be the rate-limiting step for division because it would enable PCC formation (which is rate-limiting under these conditions; above). In contrast, in slow growth conditions, where accumulation of cell material is apparently limiting irrespective of PCC formation (above), acquisition of sufficient material would activate the already assembled PCC.

Two very recent reports are fully consonant with the above suggestions.

- First, recent single cell analyses raise the possibility that inputs from both growth and the chromosome cycle are integrated to determine division timing on a single cell level and to a corresponding suggestion that division is defined by an “AND” gate which takes into account inputs from both components (Micali et al., 2018a,b). However, differently from the model proposed here, where integration of different components is upstream of initiation and division, the proposed “AND” gate integrates chromosome and divisome requirements at a later stage to actually set the timing of cell division in individual cells. We also note that the actual occurrence of cell division by our model involves not only PCC activation but the downstream events involved in implementation of septum formation, which is also sensitive to growth conditions (Coltharp et al., 2016).
- Woldringh and colleagues (Huis et al., 2018) have presented evidence that, in fast-growing cells, prominent nucleoid duality occurs earlier in larger newborn cells than in smaller newborns. At any given growth condition, larger cells accumulate mass more rapidly and thus divide sooner than smaller cells. These authors suggest that this difference in time to division is explained by the different amounts of time required for progression to duality. Modeling further suggests that the relationship between replication initiation and segregation, whose effects play out in the timing of the duality transition, can underlie variations in inter-division time under different fast growth conditions. This scenario is similar in spirit to the model presented above where (i) nucleoid duality plays a prominent role in enabling the proposed PCC development and (ii) resultant development of this chromosome-divisome complex is the rate limiting step for division in fast growth conditions.

## Author contributions

NK, KC, MW, JF, MS contributed to the concepts in this paper and to the preparation of the manuscript.

### Conflict of interest statement

The authors declare that the research was conducted in the absence of any commercial or financial relationships that could be construed as a potential conflict of interest.

## Footnotes

^1^The “progression permission” proposal outlined above is an extension of our previously described “licensing hypothesis”. The validity of that hypothesis has sometimes been questioned. However, all such statements of which we are aware are all based on fundamental misunderstanding of the basic idea. Since the same misunderstandings would pertain to the hypothesis described here, we address them directly. Two misunderstandings are most common. (1) The hypothesis is stated to be wrong because it does not accommodate effects on replication initiation timing due to changes in DnaA concentration. This is incorrect because “licensing” is, by its nature, a “permission-granting” process. It does *not* determine the timing of replication, which instead is determined during the implementation step, downstream of “licensing”, by the concentrations of DnaA-ATP, IHF, supercoiling and likely additional factors. The same considerations apply to cell division. To eliminate this confusion, we have subdivided the proposed steps into “progression permission” and “implementation”. (2) The hypothesis has been deemed invalid because replication initiation can occur in an *ftsZ* mutant, where division does not occur. This is incorrect for two reasons. (a) Cell division and licensing (progression permission) for replication initiation do not occur in series in a dependent relationship; instead, they are parallel downstream outcomes. (b) In order for replication initiation to be “licensed”, it must first be impeded; this impediment was proposed then (as above) to involve association with the cell septum; and thus, when there is no cell septum, there will be no association and thus no impediment. Therefore, the phenotype of an *ftsZ* mutant as predicted by our hypothesis is that the chromosome cycle will run free, on its own intrinsic “clock”, which is what is observed. The presentation above makes more explicit the assumptions upon which the ideas are based.
